# In vivo electrical conductivity measurement of muscle, cartilage, and peripheral nerve around knee joint using MR-electrical properties tomography

**DOI:** 10.1038/s41598-021-03928-y

**Published:** 2022-01-07

**Authors:** Ji Hyun Lee, Young Cheol Yoon, Hyun Su Kim, Jiyeong Lee, Eunju Kim, Christian Findeklee, Ulrich Katscher

**Affiliations:** 1 Department of Radiology, Samsung Medical Center, Sungkyunkwan University School of Medicine, 81 Irwon-ro, Gangnam-gu, 06351 Seoul Korea; 2 Philips Korea, Seoul, Korea; 3 Philips Research Hamburg, Hamburg, Germany

**Keywords:** Biomarkers, Medical research, Musculoskeletal system, Cartilage, Muscle

## Abstract

This study aimed to investigate whether in vivo MR-electrical properties tomography (MR-EPT) is feasible in musculoskeletal tissues by evaluating the conductivity of muscle, cartilage, and peripheral nerve around the knee joint, and to explore whether these measurements change after exercise. This prospective study was approved by the institutional review board. On February 2020, ten healthy volunteers provided written informed consent and underwent MRI of the right knee using a three-dimensional balanced steady-state free precession (bSSFP) sequence. To test the effect of loading, the subjects performed 60 squatting exercises after baseline MRI, immediately followed by post-exercise MRI with the same sequences. After reconstruction of conductivity map based on the bSSFP sequence, conductivity of muscles, cartilages, and nerves were measured. Measurements between the baseline and post-exercise MRI were compared using the paired *t*-test. Test–retest reliability for baseline conductivity was evaluated using the intraclass correlation coefficient. The baseline and post-exercise conductivity values (mean ± standard deviation) [S/m] of muscles, cartilages, and nerves were 1.73 ± 0.40 and 1.82 ± 0.50 (*p* = 0.048), 2.29 ± 0.47 and 2.51 ± 0.37 (*p* = 0.006), and 2.35 ± 0.57 and 2.36 ± 0.57 (*p* = 0.927), respectively. Intraclass correlation coefficient for the baseline conductivity of muscles, cartilages, and nerves were 0.89, 0.67, and 0.89, respectively. In conclusion, in vivo conductivity measurement of musculoskeletal tissues is feasible using MR-EPT. Conductivity of muscles and cartilages significantly changed with an overall increase after exercise.

## Introduction

Electrical conductivity is an intrinsic property of a material that represents the capability to transfer electrical current inside the medium^1^. In biological tissues, it is closely related to water content, intracellular and extracellular fluid volumes, ion concentration, and the cellular membrane permeability, thus has been considered to be a fundamental biophysical property^2,3^. As several experimental studies have shown that conductivity can reflect pathologic status as well as tissue type^4–7^, it has emerged as a potentially important tissue biomarker for clinical diagnostics^8,9^.

While previous investigations mostly relied on ex vivo measurements^4–7^, the first non-invasive in vivo assessment of conductivity was made using the electrical impedance tomography (EIT)^10^. Among multiple efforts to improve non-invasive conductivity imaging, MR-EIT provided higher spatial resolution and eliminated the need to solve the ill-posed inverse problem in EIT^11^. However, as it required injection of electrical current into the body, safety concerns were raised^9^. Another technique called magnetic induction tomography did not involve direct contact with the subject, nevertheless, this non-MRI-based method exhibited poor spatial resolution^12^.

Based on the concept of deriving conductivity from the measured MR signals^13^, another non-invasive approach termed “MR-electrical properties tomography (MR-EPT)” has been introduced by Katscher et al.^14^. They reconstructed tissue conductivity from the measured *B*_1_^+^ field generated by the radiofrequency (RF) coil. It is considered to be advantageous regarding practicability, as it can be acquired using standard MRI system with a regular coil and does not apply externally mounted electrodes or current^8^. With increasing interest, there has been continuous progress in the research of MR-EPT to validate it^15^, prove its feasibility^16^, and estimate specific absorption rate (SAR)^17^. Whilst several clinical studies have investigated MR-EPT in various organs including breast^18,19^, brain^20^, and liver^21^, the number of studies that utilized MR-EPT in musculoskeletal tissues has been limited^22^.

Thus, the purpose of this study was to investigate whether in vivo imaging is feasible in musculoskeletal tissues using MR-EPT by evaluating the conductivity of muscle, cartilage, and peripheral nerves around the knee joint. Under the hypothesis that conductivity could reflect physiologic changes, this study also sought to explore whether the conductivity of musculoskeletal tissues changes after exercise, and to interpret their changes with reference to other quantitative measurements including T2, T2* relaxation times (RT) and diffusion tensor imaging (DTI) parameters.

## Materials and methods

This prospective study was approved by the institutional review board (Samsung Medical Center, IRB File No. 2019-11-032) and registered at cris.nih.go.kr (KCT0004839). After being advised of the purpose of the study, all subjects gave written informed consent. The study was conducted in accordance with the declaration of Helsinki.

### Study population

According to a previous study^22^, the mean and standard deviation of muscle conductivity using MR-EPT has been reported to be 0.93 S/m (Siemens per meter = Ω^−1^ m^−1^) and 0.26 S/m, respectively. The sample size (*n*) needed to construct a 95% confidence interval with a margin of error of 0.17 S/m was prospectively calculated using the following equation: *n* = 1.96^2^ × SD^2^/*d*^2^, where SD and *d* are the standard deviation of muscle conductivity and the margin of error, respectively. Therefore, the sample size was calculated to be nine. Assuming dropout rate to be 10%, ten subjects were required.

Ten healthy adults were recruited who had body mass index between 18.5 and 25.0, without any discomfort in their lower extremities, previous history of internal derangement, surgery in the right knee, or contraindication to MRI such as claustrophobia.

### Magnetic resonance imaging acquisition

All volunteers underwent baseline imaging using a 3.0-T MRI scanner (Ingenia CX, Philips Medical Systems) and a 16-channel knee coil. For conductivity assessment, a three-dimensional (3D) balanced steady-state free precession (bSSFP) (balanced fast field echo [bFFE], Philips Medical Systems) sequence was chosen; this sequence was selected due to its relatively benign off-resonance behavior and robustness against unwanted phase contribution from flow, motion, and eddy current^8^. Both magnitude and phase images were obtained in the axial plane. To evaluate the test–retest reliability of conductivity, all volunteers underwent two iterations of the bSSFP sequence. Thereafter, T2 map (T2-weighted multi-echo spin-echo sequence), T2* map (T2*-weighted multi-echo fast-field echo sequence with ∆*B*_0_ correction), and DTI (single-shot echo-planar imaging) in the axial planes were acquired. T2 and T2* maps were computed using a pixel-by-pixel basis, mono-exponential, non-negative least-square fitting algorithm. For DTI, diffusion gradients were applied in six directions and diffusion encoding was done with monopolar gradient pulses. Detailed MRI parameters are summarized in Table 1.

**Table 1:** Parameters of MR sequences.

	bSSFP	T2 map	T2* map	DTI
TR (ms)	4.5302	1735.2	61.8963	3399.1
TE (ms)	2.265	13 (13, 6)*	2.83 (2.354, 15)*	61.344
Acquisition matrix	224 × 222	400 × 286	400 × 400	116 × 114
Reconstruction matrix	352 × 352	400 × 400	448 × 448	128 × 128
Pixel spacing (mm)	0.568	0.500	0.446	1.562
Field of view (cm)	20	20	20	20
Slice Thickness/gap (mm)	1/0	3/0.3	3/0.3	3/0
b values	–	–	–	0, 600
Number of averages	2	1	1	2
Number of slices	80	20	20	30
Echo train length	1	6	15	57
Parallel reduction factor	1	2	2.1	2
Flip angle (°)	30	90	25	90
Acquisition time	4 min 12 s	4 min 9 s	3 min 14 s	3 min 34 s

TR, repetition time; TE, echo time; bSSFP, balanced steady-state free precession; DTI, diffusion tensor imaging.

*Data are the first TE with ΔTE and the number of TE values in parentheses.

### Exercise protocol and post-exercise imaging

To test the effect of loading, the volunteers performed squatting exercise outside the MRI scanner after baseline imaging as described in a previous literature^23^. Standing with weight equally distributed and the feet separated shoulder width apart, they were asked to stand in a position of knee flexion at 90° bilaterally. Keeping the knees not moving past the toes, they held this position for 3 s, followed by repositioning to stand upright. They performed 60 repetitions under supervision by an observer to ensure identical exercise among the volunteers. After exercising, the volunteers were immediately repositioned on the MR table for post-exercise imaging using the same sequences and protocols as the baseline imaging, including two iterations of the bSSFP sequence. The first and second post-exercise bSSFP sequences were acquired 2 and 6 min after exercise cessation in all volunteers, respectively. Volunteers’ skin was marked under the guidance of integrated laser to maintain identical imaging position between the baseline and post-exercise imaging.

### EPT reconstruction

After data acquisition, all images were transferred to an independent workstation for analysis with manufacturer-supplied software (Extended Philips Research Software Solution [EXPRESS], Philips Healthcare). The conductivity maps were reconstructed from the phase images of the 3D bSSFP sequence. To convert the phase information to conductivity (σ), the following equation (assuming constant *B*_1_ magnitude) was used:

$$ \sigma = \Delta \Phi^{ + } /(\mu \omega ) $$

where *ω* = Larmor frequency, Δ = Laplacian operator, *μ* = magnetic permeability of tissue (assumed to be equal to magnetic permeability of free space), and *Φ*^+^  = RF transmit phase estimated as half of the measured transceive phase *Φ*^±^ (i.e., *Φ*^+^ ≈ *Φ*^±^/2)^8^. Care has to be taken that *Φ*^±^ does not show any phase wraps when applying this division by two. However, since the total phase span across the field of view has been roughly 60° for the experiments in this study, no dedicated phase unwrapping was needed. The second derivative required for Δ was performed numerically by fitting a local parabola to a number *N* of voxels (“kernel”) on each side of the respective target voxel of *Φ*^±^. A maximum number of *N* was chosen as *N*_max_ = 10 and reduced as soon as the bSSFP magnitude of a kernel voxel deviates more than 10% from the target voxel, providing a suitable trade-off between too low signal-to-noise ratio (for too small *N*) and decreased image resolution (for too large *N*) and avoids the typical boundary artifacts of EPT. As the Laplacian operator inevitably leads to noise amplification, a bilateral median filter was applied to the conductivity resulting from those equations, again applying the locally shaped kernel as done for the differentiation step. This approach has successfully been applied in previous studies for brain tumor conductivity^20,24^ and similarly also for breast tumor conductivity^19,25^.

### Image analysis

**Figure 1:**
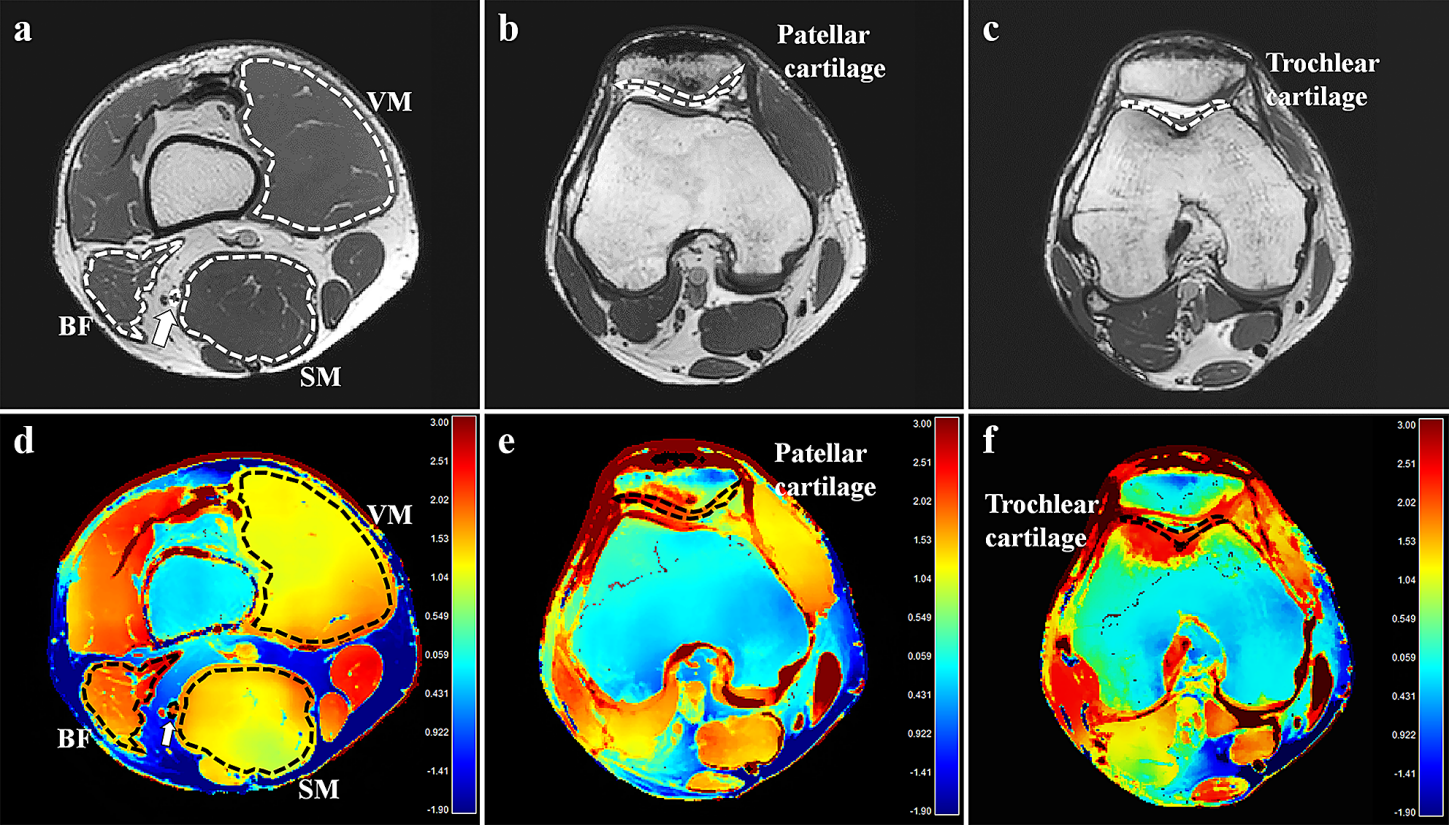
An example of conductivity analysis in the right knee of a 30-year old male volunteer. Regions of interest (ROIs) were placed in the vastus medialis (VM), semimembranosus (SM) muscles, biceps femoris muscle, including both long and short heads (BF), tibial nerve at the distal thigh level, and patellar and trochlear cartilage on the axial three-dimensional balanced fast-field echo sequence (**a**–**c**). ROIs with identical shapes, sizes, and positions were automatically generated on the conductivity map (**d**–**f**). The unit for the scale bars is S/m. Arrows, tibial nerve; dotted lines, ROIs.

Two board-certified radiologists (readers I and II; 5 and 1 years of experience in musculoskeletal imaging, respectively) analyzed the MRIs. Among T2-weighted images with six different echo times, one was selected and regarded as anatomical reference that best differentiated subchondral bone, articular cartilage, and joint fluid from the cartilage. Using the manufacturer-supplied software (Extended Philips Research Software Solution [EXPRESS], Philips), conductivities of muscles, cartilages, and nerves were measured by placing regions of interests (ROIs) on the magnitude image of the 3D bSSFP sequence using freehand drawing tools according to the following criteria (Fig. 1):

1. Muscles: At the level of the distal thigh, the vastus medialis (VM), semimembranosus muscles (SM), and biceps femoris muscle, including both long and short heads (BF) were selected because they had sufficient cross-sectional area. The ROIs were placed on each muscle so that the boundaries were within 1–2 mm from the muscle fascia.
2. Cartilages: Axial planes that revealed the maximum thickness of the patellar and trochlear cartilages were selected for evaluation. The ROIs were placed to cover the entire cartilage, encompassing both deep and superficial cartilage, and medial and lateral facets; joint fluid or subchondral bone were carefully avoided.
3. Nerve: At the same axial plane as that for muscle analysis, the ROIs were carefully drawn to fall within the visualized boundary of the tibial nerve, excluding the perineural vessels.

Dedicated software (IntelliSpace Portal, version 10.0; Philips) was used for image processing and ROI placement on the T2, T2* maps, and DTI using the same criteria and anatomical reference images. The mean T2 and T2* RTs, fractional anisotropy (FA), and mean diffusivity (MD) of the same muscles, cartilages, and nerves were measured at the corresponding areas.

### Statistical analysis

Paired *t*-test was used to explore if the post-exercise values varied from the baseline imaging. Test–retest reliability between conductivities obtained from the 1st and 2nd baseline bSSFP sequences by reader I and interobserver agreement between the two readers were assessed using the intraclass correlation coefficient (ICC). An ICC of 1.0 was considered to represent perfect agreement; 0.81–0.99, almost perfect agreement; 0.61–0.80, substantial agreement; 0.41–0.60, moderate agreement; 0.21–0.40, fair agreement; and 0.20 or less, slight agreement^26^. All statistical analyses were performed using MedCalc version 19.0.7 (MedCalc Software Ltd). *p* values less than 0.05 were considered to indicate statistical significance.

## Results

The subjects constituted of five men and five women volunteers, ranging in age from 24 to 30 years (26.8 ± 1.8 years). The patellar and trochlear cartilages in two volunteers, were excluded on second baseline imaging due to severe artifacts. The duration of exercise ranged from 3 min 56 s to 7 min 51 s (6 min 11 s ± 1 min 17 s).

### Interobserver agreement and test–retest reliability

Because there was almost perfect interobserver agreement on measurements of conductivity in muscles, cartilages, and nerves, data obtained by one of the readers were used. The result of the test–retest reliability of the baseline conductivity was almost perfect in all muscles and the tibial nerve. Cartilages tended to show lower test–retest reliability, which were regarded as substantial (Table 2).

**Table 2:** Test–retest reliability and interobserver agreement for conductivity measurement.

	Test–retest reliability	Interobserver agreement
**All muscles**	0.89 (0.79–0.95)	0.98 (0.97–0.98)
VM muscle	0.80 (0.41–0.95)	0.96 (0.93–0.98)
SM muscle	0.96 (0.84–0.99)	0.97 (0.95–0.98)
BF muscle	0.84 (0.51–0.96)	0.99 (0.97–0.99)
**All cartilages**	0.67 (0.28–0.87)	0.99 (0.98–0.99)
Patellar cartilage	0.71 (0.09–0.94)	0.98 (0.97–0.99)
Trochlear cartilage	0.64 (− 0.05–0.91)	0.99 (0.97–0.99)
**Tibial nerve**	0.89 (0.63–0.97)	0.92 (0.85–0.96)

Data are intraclass correlation coefficients and 95% confidence intervals in the parentheses.

CI, confidence interval; VM, vastus medialis; SM, semimembranosus; BF, biceps femoris; and ICC, intraclass correlation coefficient.

### Baseline conductivity measurements

The range of baseline conductivity was as follows: all muscles, 1.03–2.58 S/m; VM muscle, 1.03–1.95 S/m; SM muscle, 1.15–2.48 S/m; BF muscle, 1.45–2.58 S/m; all cartilages, 1.12–2.98 S/m; patellar cartilage, 1.11–2.80 S/m; trochlear cartilage, 1.51–2.98 S/m; tibial nerve, 1.47–3.00 S/m.

### Post-exercise changes of conductivity

**Figure 2:**
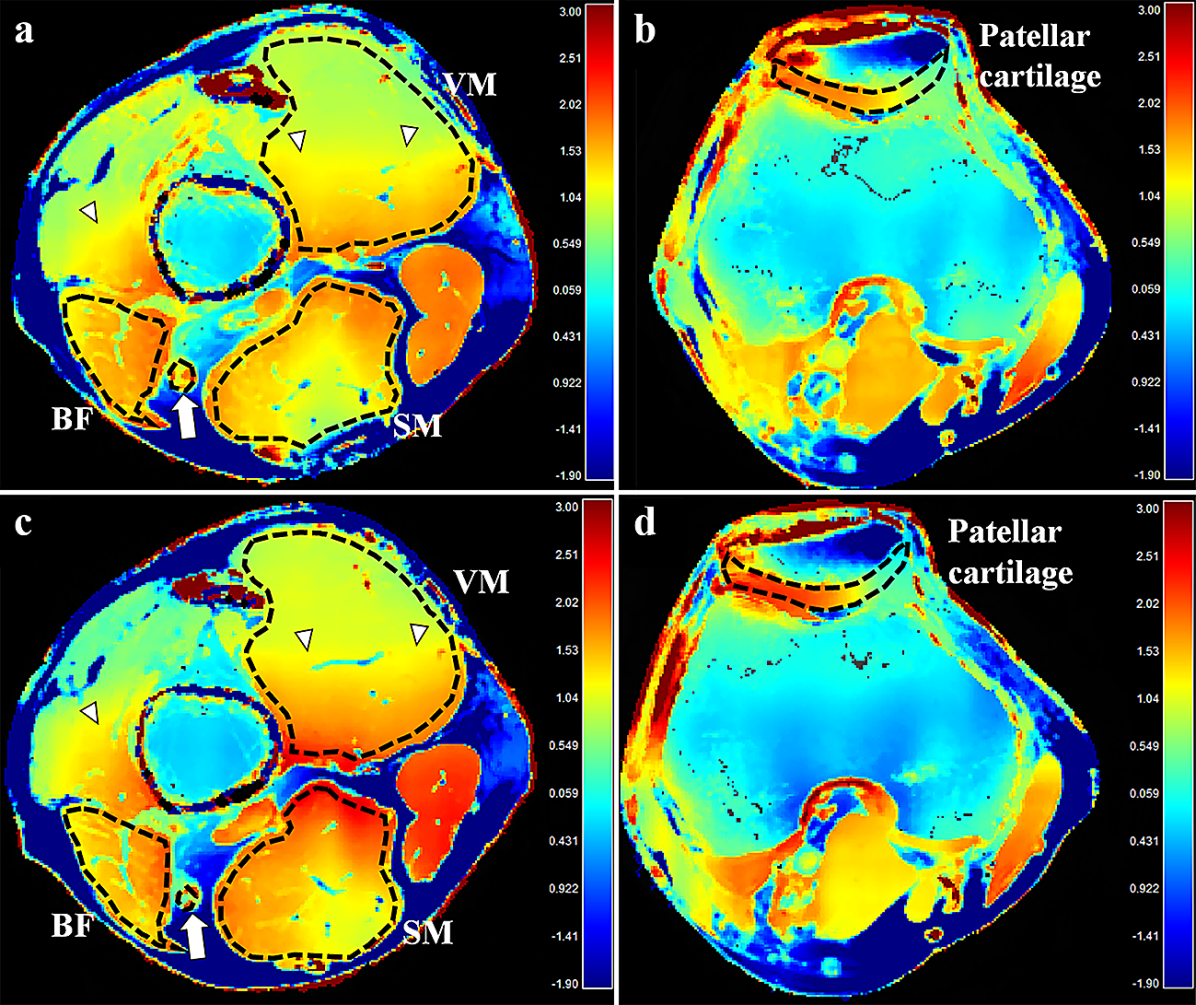
Baseline (**a**, **b**) and the post-exercise conductivity map (**c**, **d**) in a 28-year old male volunteer. Compared to the baseline, conductivity of the vastus medialis muscles and patellar cartilage increased from 1.033 to 1.188 S/m, and from 1.119 to 1.450 S/m, respectively. *B*_1_ inhomogeneity is noted across field of view, shown as demarcated areas with increased conductivity in red-colored zones (arrowheads). The unit for the scale bars is S/m. BF, biceps femoris muscle; SM, semimembranosus muscle; VM, vastus medialis muscle; arrows, tibial nerve; dotted lines, ROIs.

**Figure 3:**
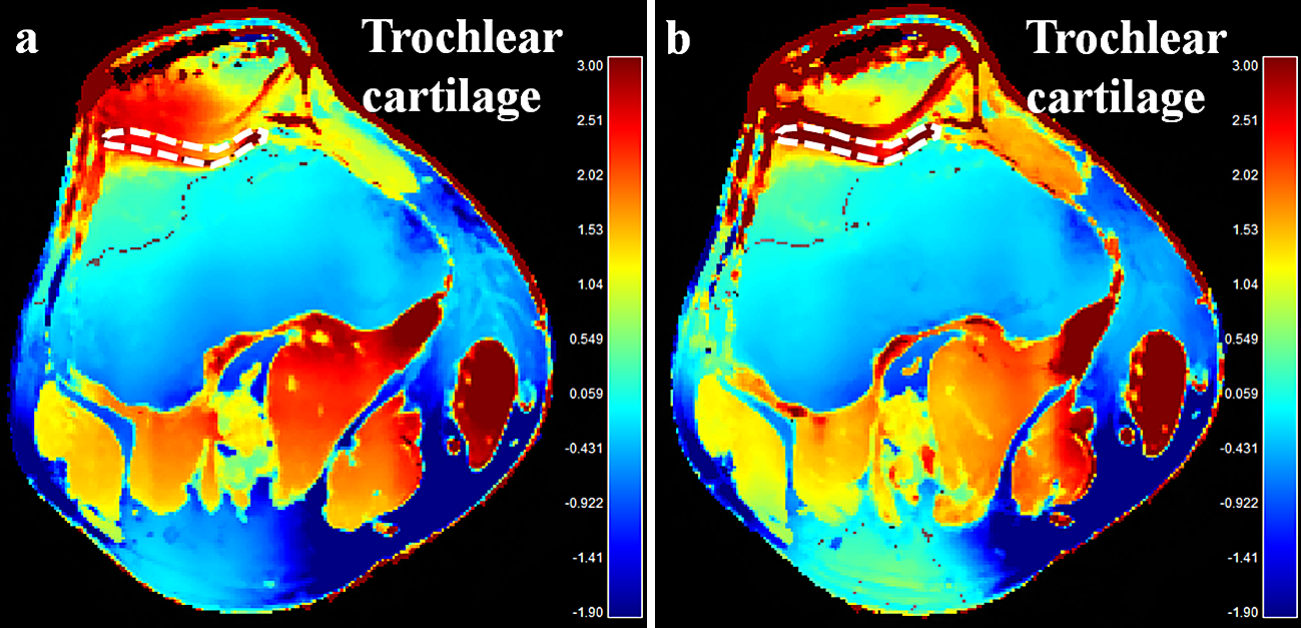
Baseline (**a**) and the post-exercise conductivity map (**b**) in a 28-year old male volunteer. Compared to the baseline, conductivity of the trochlear cartilage increased from 1.887 to 2.539 S/m. The unit for the scale bars is S/m. dotted lines, ROIs.

**Figure 4:**
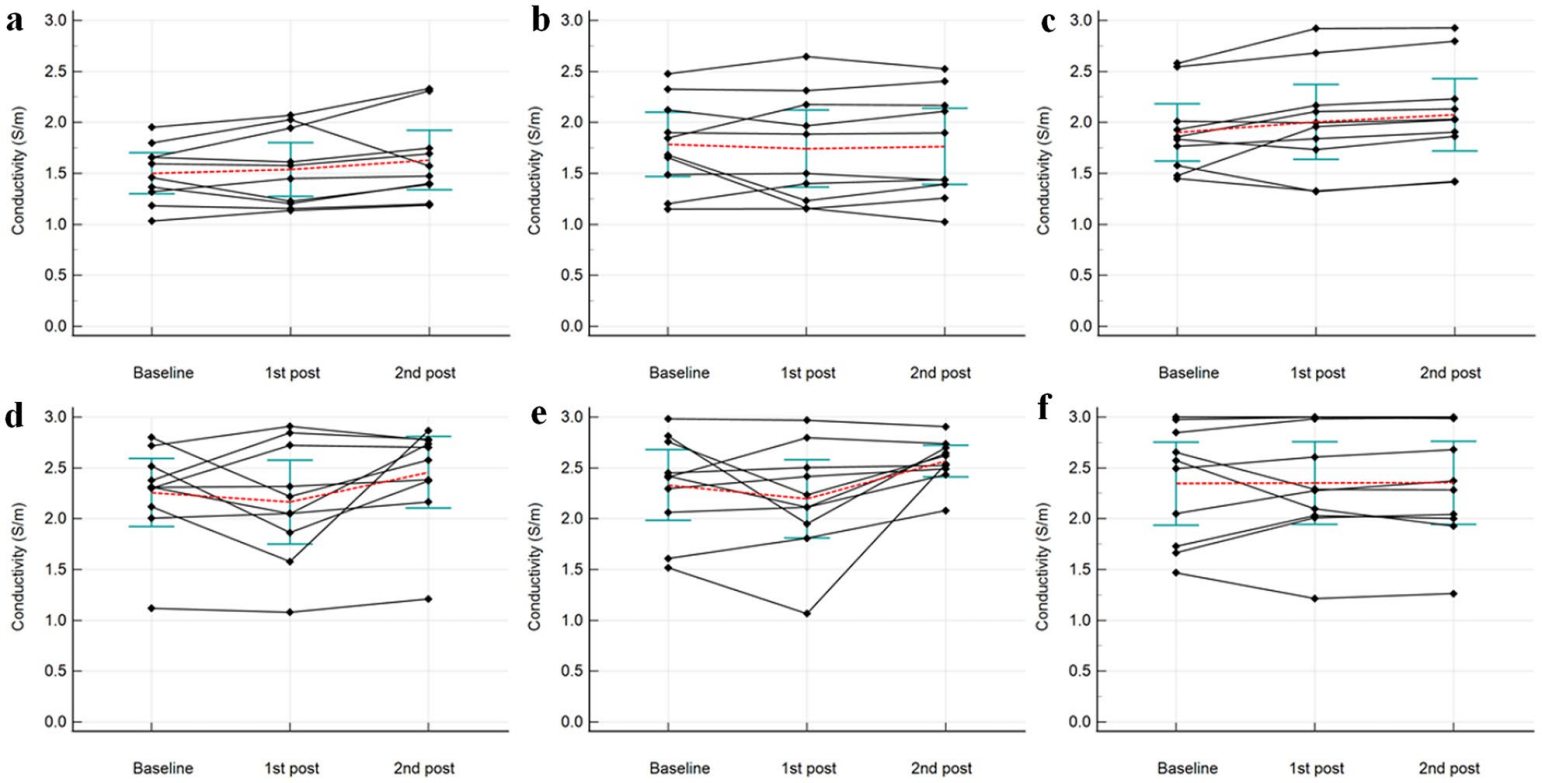
Graphs showing change of conductivity of vastus medialis muscle (**a**), semimembranosus muscle (**b**), biceps femoris muscle (**c**), patellar cartilage (**d**), trochlear cartilage (**e**), and tibial nerve (**f**) for each volunteer. The mean values are represented by red dotted lines, 95% confidence intervals of mean values by blue green bars, respectively.

Means and standard deviations of conductivity in muscles, cartilages, and nerves during the baseline, first, and second post-exercise imaging are summarized in Table 3. Conductivity of all muscles, the BF muscle, and all cartilages on the second post-exercise imaging significantly changed by + 5.41 ± 14.36% (*p* = 0.048), + 9.05 ± 11.07% (*p* = 0.029), and + 9.49 ± 13.69% (*p* = 0.006), respectively [mean ± standard deviation], with an overall increase from the baseline; conductivity of the VM muscle, patellar, and trochlear cartilages tended to increase on the second post-exercise imaging, which changed by + 8.59 ± 16.12% (*p* = 0.126), + 8.84 ± 4.13% (*p* = 0.061), and + 10.12 ± 4.71% (*p* = 0.060), respectively (Figs. 2, 3, 4). All conductivities of the first post-exercise imaging and conductivity of the tibial nerve on the second post-exercise imaging were not significantly different from the baseline imaging.

**Table 3:** Conductivity before and after exercise.

	Baseline	1st Post-exercise	95% CI of differences*	*p* value*	2nd Post-exercise	95% CI of differences^†^	*p* value^†^
**All muscles**	1.73 ± 0.40	1.76 ± 0.50	− 0.05 to + 0.12	0.428	1.82 ± 0.50	+ 0.00 to + 0.19	0.048
VM muscle	1.50 ± 0.28	1.54 ± 0.37	− 0.08 to + 0.16	0.482	1.63 ± 0.41	− 0.04 to + 0.30	0.126
SM muscle	1.79 ± 0.44	1.74 ± 0.53	− 0.23 to + 0.15	0.626	1.76 ± 0.52	− 0.21 to + 0.17	0.819
BF muscle	1.90 ± 0.39	2.00 ± 0.51	− 0.06 to + 0.27	0.191	2.08 ± 0.50	+ 0.02 to + 0.32	0.029
**All cartilages**	2.29 ± 0.47	2.18 ± 0.54	− 0.29 to + 0.07	0.202	2.51 ± 0.37	+ 0.07 to + 0.36	0.006
Patellar cartilage	2.26 ± 0.47	2.16 ± 0.58	− 0.38 to + 0.20	0.483	2.46 ± 0.49	− 0.01 to + 0.41	0.061
Trochlear cartilage	2.33 ± 0.49	2.20 ± 0.54	− 0.41 to + 0.14	0.299	2.57 ± 0.22	− 0.01 to + 0.48	0.060
**Tibial nerve**	2.35 ± 0.57	2.35 ± 0.57	− 0.20 to + 0.21	0.953	2.36 ± 0.57	− 0.22 to + 0.24	0.927

Data are mean ± standard deviation (S/m).

VM, vastus medialis; SM, semimembranosus; BF, biceps femoris; and CI, confidence interval.

*Between the baseline and 1st post-exercise MRI.

^†^Between the baseline and 2nd post-exercise MRI.

### Post-exercise changes of other quantitative parameters

After exercise, the T2 RT, T2* RT, FA, and MD of the VM muscle changed by + 17.54 ± 11.10% (*p* < 0.001), + 19.89 ± 8.37% (*p* < 0.001), + 5.98 ± 6.11% (*p* = 0.013), and + 14.89 ± 4.42% (*p* < 0.001), respectively, with an overall increase compared to the baseline. In contrast, the BF muscle showed overall post-exercise decrease of the T2 RT, T2* RT, and MD, which changed by − 3.82 ± 4.39% (*p* = 0.022), − 3.79 ± 4.66% (*p* = 0.030), and − 3.59 ± 2.93% (*p* = 0.004) from the baseline, respectively. Regarding all muscles, overall post-exercise increase was observed in the T2* RT and FA, which changed by + 4.34 ± 11.97% (*p* = 0.018) and + 3.52 ± 6.72% (*p* = 0.012) from the baseline, respectively. No other significant differences in the T2 RT, T2* RT, FA, and MD were noted between the baseline and post-exercise imaging (Table 4).

**Table 4:** Quantitative measurements before and after exercise.

	Baseline	Post-exercise	95% CI of difference*	*p* value*
**T2 RT (ms)**				
All muscles	38.39 ± 3.17	39.91 ± 4.29	− 0.16 to + 3.20	0.074
VM muscle	37.35 ± 1.71	43.90 ± 4.03	+ 3.58 to + 9.52	< 0.001
SM muscle	35.89 ± 1.01	35.50 ± 1.04	− 1.24 to + 0.46	0.329
BF muscle	41.93 ± 2.52	40.33 ± 1.57	− 2.92 to − 0.28	0.022
All cartilages	42.80 ± 7.71	42.06 ± 6.98	− 1.81 to + 0.34	0.168
Patellar cartilage	37.49 ± 6.04	37.27 ± 5.58	− 1.17 to + 0.73	0.612
Trochlear cartilage	48.10 ± 5.14	46.85 ± 4.56	− 3.36 to + 0.86	0.214
Tibial nerve	54.82 ± 5.57	53.93 ± 4.75	− 2.38 to + 0.60	0.208
T2* RT (ms)				
All muscles	26.13 ± 1.36	27.61 ± 3.66	+ 0.28 to + 2.70	0.018
VM muscle	26.80 ± 0.92	32.13 ± 2.11	+ 3.73 to + 6.93	< 0.001
SM muscle	25.75 ± 1.24	25.86 ± 1.27	− 0.92 to + 1.14	0.815
BF muscle	25.83 ± 1.67	24.85 ± 1.55	− 1.84 to − 0.12	0.030
All cartilages	31.56 ± 6.36	31.27 ± 6.15	− 1.26 to + 0.68	0.538
Patellar cartilage	27.05 ± 4.89	26.80 ± 4.82	− 1.45 to + 0.95	0.648
Trochlear cartilage	36.07 ± 4.03	35.74 ± 3.50	− 2.12 to + 1.46	0.686
Tibial nerve	25.56 ± 4.67	25.17 ± 5.51	− 2.02 to + 1.24	0.601
**FA**				
All muscles	0.247 ± 0.035	0.255 ± 0.034	+ 0.002 to + 0.014	0.012
VM muscle	0.234 ± 0.016	0.248 ± 0.017	+ 0.004 to + 0.024	0.013
SM muscle	0.219 ± 0.017	0.227 ± 0.023	− 0.002 to + 0.018	0.104
BF muscle	0.287 ± 0.021	0.289 ± 0.027	− 0.012 to + 0.016	0.751
All cartilages	0.134 ± 0.042	0.124 ± 0.030	− 0.028 to + 0.009	0.298
Patellar cartilage	0.129 ± 0.038	0.113 ± 0.017	− 0.039 to + 0.007	0.149
Trochlear cartilage	0.138 ± 0.047	0.135 ± 0.036	− 0.037 to + 0.031	0.844
Tibial nerve	0.539 ± 0.011	0.552 ± 0.048	− 0.054 to + 0.080	0.672
**MD (× 10**^**–3**^** mm**^**2**^**/s)**				
All muscles	1.57 ± 0.08	1.59 ± 0.26	− 0.06 to + 0.11	0.570
VM muscle	1.63 ± 0.06	1.87 ± 0.05	+ 0.19 to + 0.29	< 0.001
SM muscle	1.52 ± 0.06	1.41 ± 0.27	− 0.33 to + 0.10	0.269
BF muscle	1.56 ± 0.10	1.51 ± 0.08	− 0.09 to − 0.02	0.004
All cartilages	1.68 ± 0.14	1.66 ± 0.23	− 0.15 to + 0.09	0.645
Patellar cartilage	1.69 ± 0.14	1.62 ± 0.30	− 0.30 to + 0.16	0.530
Trochlear cartilage	1.68 ± 0.14	1.70 ± 0.12	− 0.10 to + 0.13	0.792
Tibial nerve	1.15 ± 0.16	1.08 ± 0.16	− 0.19 to + 0.05	0.246

Data are mean ± standard deviation.

VM, vastus medialis; SM, semimembranosus; BF, biceps femoris; RT, relaxation time; FA, fractional anisotropy; MD, mean diffusivity; and CI, confidence interval.

*Between the baseline and post-exercise MRI.

## Discussion

This study measured in vivo conductivity of muscles, cartilages, and nerves around the knee joint in normal volunteers using MR-EPT and showed its feasibility. Additionally, this study revealed that post-exercise conductivity significantly changed with a tendency to increase in muscles and cartilages at 6 min after exercise cessation.

In previous animal studies, results of muscle conductivity assessment observed a large variation ranging between 0.56 and 1.05 S/m^27–29^, probably due to various species and study conditions. In human subjects, muscle conductivity has been reported to be 0.62 S/m^30^ and 0.93 S/m^22^. In ex vivo studies, conductivity of cartilages and nerves were reported to be 0.51–1.14 S/m^29,31^ and 0.39 S/m^29^, respectively. Though different experimental conditions make direct comparison difficult, the conductivity values of our study were higher than those of previous investigations.

Compared to the conductivity maps in previous studies on MR-EPT^18,20^, maps in this study were suspected to suffer from *B*_1_ inhomogeneity. The bSSFP sequence is known to have relative robustness against *B*_0_ inhomogeneity, as long as banding artifacts from signal voids are avoided, and in fact *B*_0_ inhomogeneities are too small to produce those banding artifacts in the knee images of this study; yet, conductivity reconstruction is vulnerable to *B*_1_ inhomogeneity^8^. Conductivity reconstruction as applied in this study is based on *B*_1_ phase only, i.e., based on the assumption of a constant *B*_1_ magnitude. It has been reported that a violation of this assumption leads to an artificial increase of reconstructed conductivity, contributing to the observed high conductivity values^32^. A phantom simulation as described in the Supplementary material underlines this effect, leading to roughly twice the expected conductivity as a consequence of the EPT assumptions applied. A future study shall include the additional measurement of *B*_1_ magnitude to exclude this artificial conductivity increase. Nevertheless, as *B*_1_ inhomogeneity is similar for all scans in this study, it can be expected that the observed exercise-induced increase of conductivity is not affected by this issue. Furthermore, this study adopted the so-called “transceive phase assumption”, estimating the absolute *B*_1_^+^ phase *Φ*^+^ to be half of the transceive phase *Φ*^±^ as the *B*_1_^+^ phase is not directly measurable by standard MR sequences^15,32,33^. Even though it has been widely used, violation against this assumption is thought to lower the accuracy of conductivity evaluation. Phase error has been reported to be larger at the peripheral regions indicating that the merit of this assumption deteriorates^16,34^. In addition, geometric asymmetry may lead to unequal contributions of the transmission and reception process in the total transceive phase^33^. It can also be prone to inaccuracies when imaging subjects with asymmetrical electrical properties^35^. In this context, substantial phase error might have been produced in this study. As extremities are hardly scanned in the isocenter area and are inherently asymmetric with right- or left-sided predilection, this phase error could be regarded as one of the major problems to be resolved for implementing MR-EPT in musculoskeletal imaging. Given that the previous study^22^ from which we estimated the sample size examined pelvic region, wide range of conductivity with relatively large SD in our study also might be explained by aforementioned reasons. Despite of previous study that validated MR-EPT^15^, our study suggests that it may be vulnerable to reconstruction error in musculoskeletal regions requiring further validation.

The post-exercise T2 and T2* RTs, FA, and MD significantly increased in the VM muscle, which was comparable to the findings of previous studies^36,37^. Osmotic shift, water accumulation, increase in volume of intracellular space of perfused muscle and intracellular acidification increases T2 RT^36–40^, whereas blood inflow exceeding oxygen extraction fraction increases T2* RT^36,41^. For DTI parameters, tightening of muscle fibers and changes in intra- and extracellular volume, pseudodiffusion due to increased blood volume are suggested to increase FA and MD, respectively^40,42^. In contrast, the post-exercise T2, T2* RT, and MD of the BF muscle decreased in this study, contrary to the VM muscle. As squatting elicits greater activity of quadriceps relative to other muscles^43^, decreased T2, T2* RT, and MD may imply compensatory vasoconstriction in the BF muscle.

On the other hand, conductivity tended to increase in both VM and BF muscles and cartilages on the second post-exercise MRI compared to the baseline. While conductivity is affected by both water content and tissue sodium concentration^44^, increased conductivity could not be explained by increased water content as T2 RT were decreased in the BF muscle and not changed in the cartilages. Thus, it could be reasonable to presume that increased conductivity may reflect increased tissue sodium concentration, which probably occurs several minutes after the exercise cessation rather than immediate post-exercise period. However, considering that the duration of exercise varied between the volunteers and that there was a difference in the pattern of change in conductivity after exercise as shown in Fig. 4, additional research is required to prove the timing of change in conductivity after exercise. In this context, exercise-induced time course changes of conductivity using a rapid sequential acquisition of MR-EPT would be another interesting topic of future investigation.

Our result showing changes of conductivity in muscle is partially comparable to those of previous studies reporting signal intensity increase of muscle after exercise using sodium MRI^45,46^. Whereas sodium MRI is still challenging due to limitations in hardware and software, MR-EPT is advantageous as it uses standard MR scanners. Considering usefulness of sodium MRI in evaluating early cartilage degeneration^47^, it could be reasonable to consider cartilage imaging as one of the promising applications of MR-EPT in the musculoskeletal regions. However, conductivity of the SM muscle did not show tendency of post-exercise increase. Although we presumed that no significant physiologic change occurred in the SM muscle after exercise considering all the other parameters were also not changed, the basis of this result remains unclear. Furthermore, the fact that the differences between baseline and post-exercise conductivity in muscles and cartilages were significant only when they were grouped except for the BF muscle requires future investigation to elucidate its true significance. In addition to the technical issues regarding *B*_1_ inhomogeneity, relatively low test–retest reliability in the cartilage possibly due to their small size and curved shape is another hurdle that should be overcome.

This study had several limitations. First, the MR-EPT technique was not validated in the musculoskeletal regions. Validation using a knee-sized phantom and conductivity probe could help proving its reliability in musculoskeletal imaging. Second, substantial inhomogeneity was noted in the conductivity maps with regions of increased conductivity, as described above. Further optimization would be mandatory, which may provide conductivity values closer to the values reported in previous investigations. Third, the time intervals between exercise cessation and acquisition of each sequence for quantitative measurements were variable. The duration of exercise was also variable among the volunteers, which also may have influenced the study results particularly in post-exercise changes of conductivity. Fourth, misalignment of knee joints between the baseline and post-exercise scans could have been possible. Intelligent software which automatically plans the scanning geometries would be helpful, in addition to our effort using skin marking and integrated laser. Finally, temperature measurements of target tissues were not performed, assuming them to be 37 °C for both baseline and post-exercise MRI. As temperature coefficient of conductivity variation is about 2%/°C^48^, change of temperature after exercise may have affected our study results.

In conclusion, in vivo conductivity measurement was feasible in musculoskeletal tissues using MR-EPT around the knee joint. Although technological hurdles should be overcome that might result in artificial increase of conductivity values and relatively poor reproducibility of cartilage conductivity, significant post-exercise change in conductivity may suggest its potential as a biomarker to reflect physiologic status of musculoskeletal tissues. Further clinical studies with validation based on this initial experience of MR-EPT would be mandatory to elucidate true significance of conductivity in musculoskeletal imaging and to expand its applications particularly to indirect assessment of tissue sodium concentration.

## Supplementary Information

Supplementary Information.

## Abbreviations

EIT: Electrical impedance tomography
EPT: Electrical properties tomography
RF: Radiofrequency
SAR: Specific absorption rate
RT: Relaxation time
DTI: Diffusion tensor imaging
3D: Three-dimensional
bSSFP: Balanced steady-state free precession
bFFE: Balanced fast field echo
ROI: Region of interest
VM: Vastus medialis
SM: Semimembranosus
BF: Biceps femoris
FA: Fractional anisotropy
MD: Mean diffusivity
ICC: Intraclass correlation coefficient
