# Supplementary material for: In vivo electrical conductivity measurement of muscle, cartilage, and peripheral nerve around knee joint using MR-electrical properties tomography
Source: Sci Rep. 2022 Jan 7;12:73. doi: 10.1038/s41598-021-03928-y (PMC8741940; doi:10.1038/s41598-021-03928-y)
Supplement: Supplementary file 1 — Supplementary Information. [file 41598_2021_3928_MOESM1_ESM.docx]

**Supplementary material**

The conductivity reconstruction as applied in this study uses two major assumptions: (a) that the *B*_1_ magnitude is constant, (b) that the transmit phase equals half the transceive phase. These two assumptions have been investigated in the framework of a phantom simulation described in this Supplementary material.

Electromagnetic fields were calculated with the software package “CONCEPT II” (Technical University Hamburg-Harburg, Department of Theoretical Electrical Engineering, Germany), which is a numerical simulation program of electromagnetic parameters on metallic structures and homogeneous media based upon electric-field integral equations and the method of moments. A quadrature body coil with 16 rods, diameter = 62 cm, and length = 60 cm has been modelled at 128 MHz, together with two non-concentric cylinders serving as simplified leg model (*ε*_r_ = 60, *σ* = 1.0/0.5 S/m for inner/outer cylinder). The central axis of the inner cylinder was aligned with the central axis of the quadrature body coil to obtain an off-isocenter situation. The same conductivity reconstruction (i.e., the same software tool EXPRESS) was used as for the *in vivo* scans.

Fig. A1 compares reconstructed conductivity with and without the two assumptions listed above. Without assumptions, the mean reconstruction error is 0.03 S/m. With assumptions, the mean reconstruction error is 0.19 S/m. Particularly, the reconstructed conductivity in the periphery of the outer cylinder goes up to roughly 0.9 S/m instead the expected 0.5 S/m. This finding of roughly twice the expected conductivity coincides with the *in vivo* findings of this study.


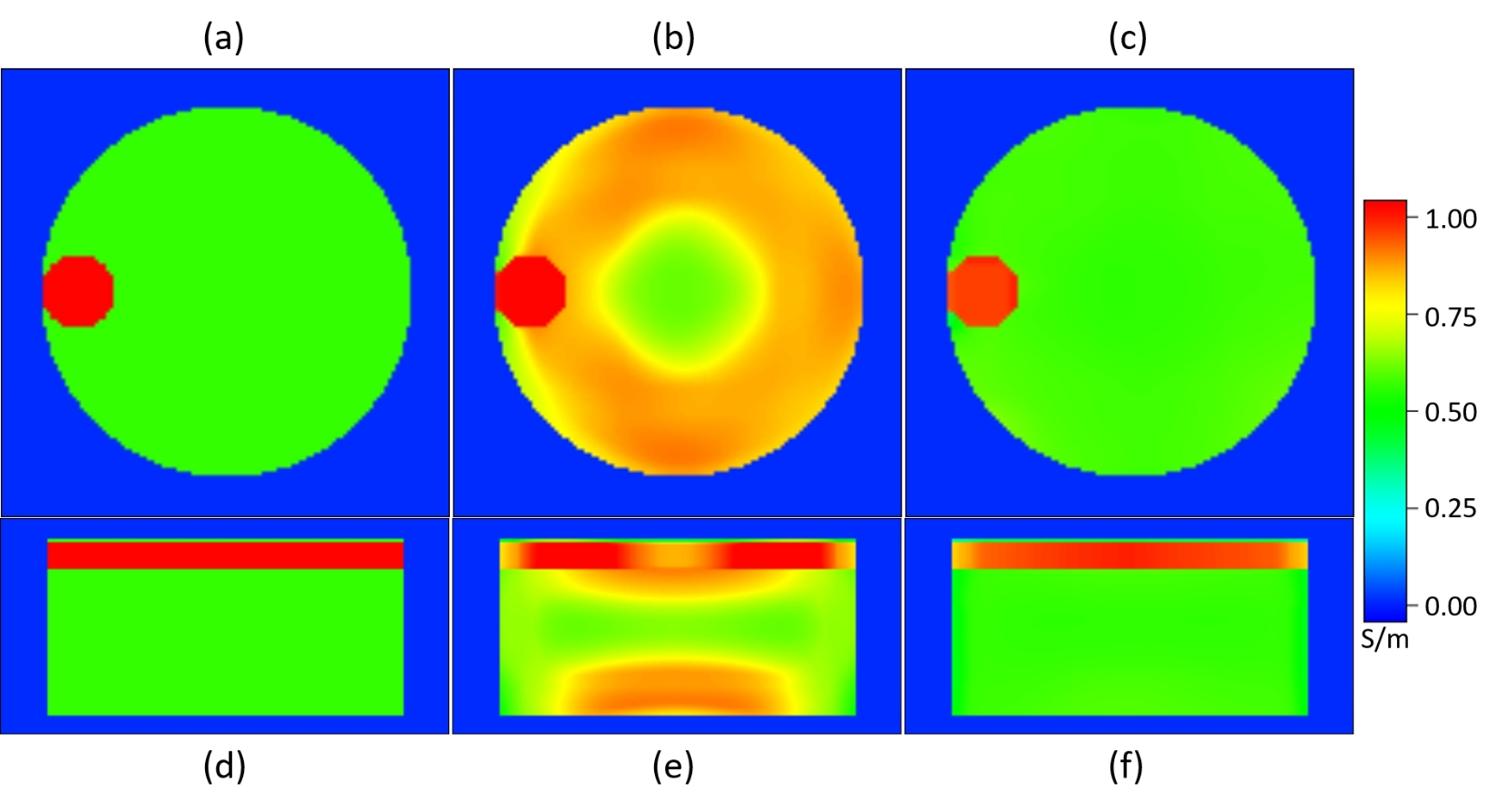


**Fig. A1:** Phantom simulation. Conductivity reconstructed with (b,e) and without (c,f) EPT assumptions are compared with the expected conductivity (a,d). Axial slices are shown in (a-c) and coronal slices are shown in (d-f). With no assumptions applied, the reconstructed conductivity is close to the expected conductivity. With assumptions applied, the reconstructed conductivity is up to twice the expected conductivity, as found in the *in vivo* experiments of this study.
